# Bacteriophage P1 protein Icd inhibits bacterial division by targeting FtsZ

**DOI:** 10.3389/fmicb.2025.1533694

**Published:** 2025-02-26

**Authors:** Kairui Zhao, Shuheng Du, Linlin Tian, Shenping Wang, Runqin Shi, Haiyu Sun, Yao Zhou, Chenhao Huang, Yanmei Sun, Shiwei Wang, Yaodong Chen

**Affiliations:** ^1^Key Laboratory of Resources Biology and Biotechnology in Western China, Ministry of Education, College of Life Sciences, Northwest University, Xi’an, China; ^2^Provincial Key Laboratory of Biotechnology of Shaanxi Province, Northwest University, Xi’an, China

**Keywords:** bacteriophage P1, ICD, bacterial cell division, FtsZ, phage-host

## Abstract

The study of bacteriophage (phage) gene products and their effects on the host helps to better understand the phage-host relationship and provides clues for the development of new antimicrobial proteins. In this study, we focused on a small protein named Icd with 73 amino acids from phage P1. It inhibits the growth of *Escherichia coli* and rapidly blocks the formation of Z-ring. The results of bacterial two-hybrid and pull-down experiments showed that Icd directly targets FtsZ, a key protein in bacterial division. Furthermore, we identified the core region of Icd as amino acids 12–51; this 40-amino acid protein had similar antibacterial activity to the full-length Icd, inhibiting bacterial growth and division.

## Introduction

The continued increase in pathogenic microorganisms and their drug-resistant strains pose a serious threat to human health (Munita and Arias, 2016). Drug-resistant bacteria are found in various human habitats, and the growing urgency has led to research into new antibiotics, including new antimicrobial proteins (AMP), as effective solutions.

FtsZ is an attractive target for developing novel antibiotics (Ur Rahman et al., 2020; Kusuma et al., 2019), which is conserved in most bacteria and archaea, and plays a crucial role in most pathogenic microorganisms. FtsZ is essential for bacterial division (Haeusser and Margolin, 2016; McQuillen and Xiao, 2020). It was the first identified bacterial tubulin homolog (Nogales et al., 1998; Oliva et al., 2004) with the ability to hydrolyze GTP and self-polymerize into mostly single highly dynamic protofilaments (Chen and Erickson, 2005; Chen and Erickson, 2009). FtsZ serves as a scaffold for the Z ring that assembles at the bacterial division site, recruiting dozens of proteins to form the mature divisome to complete division (McQuillen and Xiao, 2020; Bi and Lutkenhaus, 1991). Z-ring *in vivo* are very dynamic, driven by the hydrolysis of GTP by FtsZ (Stricker et al., 2002), which is consistent with the highly dynamic characteristics of FtsZ filaments (Chen and Erickson, 2005; Chen and Erickson, 2009). Recent studies have revealed that they exhibit a treadmilling pattern, adding subunits to one end of the FtsZ filaments and releasing them from another end (Bisson-Filho et al., 2017; Yang et al., 2017; McCausland et al., 2021). The dynamic Z ring not only recruits proteins to form the bacterial divisome but also directs peptidoglycan synthesis for the new cell wall (McQuillen and Xiao, 2020; Yang et al., 2021). Additionally, the contraction of the Z-ring may generate the force needed to pull the membrane inward and combine with the synthesis of the new cell wall to complete division (Osawa et al., 2008; Osawa and Erickson, 2011; Erickson and Osawa, 2017).

The gene products of phages have been extensively studied, and some have been shown to inhibit the growth and division of host bacteria by targeting proteins with important physiological function, especially bacterial cell division. Kil, a small polypeptide from phage *λ*, interferes with the formation of Z-ring in *Escherichia coli*, preventing bacterial division and causing continuous elongation (Conter et al., 1996; Haeusser et al., 2014; Hernandez-Rocamora et al., 2015). Similarly, the key bacterial division protein FtsZ has been confirmed as the target of Gp0.4 (Kiro et al., 2013; Simpkin Adam and Rigden, 2016), a gene product of phage T7, and the small polypeptide Hdi from phage T5 (Mahata et al., 2023). Simpkin Adam and Rigden (2016) predicted the structure of Gp0.4 and FtsZ, constructing a “U” model that serves as a representative for Gp0.4 analysis. Bioinformatics analysis revealed that the two *α*-helices of Gp0.4 could bind to the helices 1, 5, and 7 of the FtsZ protein. Phage peptides do not only target FtsZ proteins. De Smet et al. (2021) reported that Lgy, an early gene of *Pseudomonas aeruginosa* phage LUZ24 inhibits the DNA gyrase, thereby preventing colony formation and causing severe filamentous growth (Wagemans et al., 2015). Xu et al. (2024) discovered that protein Gp11 of *Staphylococcus aureus* phage ΦNM1 blocks cell division by inhibiting peptidoglycan biosynthesis. In addition, it has been reported that some bacteriophage proteins, such as the protein p56 of *Bacillus subtilis* phage phi29 and the UGI proteins of *B. subtilis* phages PBS-1 and PBS-2, utilize negatively charged amino acids to mimic the charge distribution of DNA and bind to uracil-DNA glycosylase to inhibit its activity (Serrano-Heras et al., 2007; Mol et al., 1995; Pearl and Savva, 1995; Savva and Pearl, 1995). Interestingly, this type of uracil-DNA glycosylase inhibitor is also present in bacteria, and the *S. aureus* protein SAUGI is the first discovered in other than bacteriophages (Wang et al., 2014).

P1 phage is a temperate phage with a double-stranded DNA genome of approximately 49.8 kb, comprising at least 119 genes (Gonzales et al., 2022). Early studies revealed that Icd (Interference with Cell Division) of phage P1 can disrupt host division (Riedel et al., 1993). This small protein consists of 73 amino acids with a molecular weight of 7.3 kDa, but its target has not yet been determined.

In this study, we found that Icd caused abnormal localization of FtsZ *in vivo* and determined, through bacterial two-hybrid and pull-down assays, that Icd inhibited bacterial division by directly binding to FtsZ. We also discovered that amino acids 12–51 of Icd was the core region which can effectively inhibit bacterial division.

## Materials and methods

### Bacterial strains, plasmids, and growth conditions

The strains were grown on LB or LB agar and cultured at 37°C under oxic conditions. Liquid cultures were incubated at 37°C in an oxic environment at 220 rpm. Plasmids were constructed using standard molecular biology techniques. Templates, primers, and enzymes were added to the PCR system to amplify the DNA fragments. The DNA was digested and ligated according to the reagent instructions, and the correct recombinant plasmid was screened through colony verification and confirmed by sequencing. The strains and plasmids used in this study are listed in Supplementary Table S1, and the primers are listed in Supplementary Table S2. The dual expression plasmid pBAD22a- *ftsZ-icd* was constructed, in which *ftsZ* and *icd* genes had their own ribosome binding site (rbs).

### *In vivo* toxicity analysis

The monoclonal *E. coli* containing the pBAD22a plasmid was inoculated into 5 mL of LB liquid medium with the appropriate antibiotics. The spectrophotometer was calibrated, and once the bacterial culture reached an OD_600_ of approximately 0.4, arabinose was added to a final concentration of 0.2% to induce gene expression for 3 h. The sample with 10 μL of the bacterial solution was then taken, spotted onto LB agar, and covered with a coverslip for observation under a microscope. Cell lengths were measured for each mutant, and the results were reported as the mean ± SD. Additionally, we spotted 5 μL of dilutions from a dilution series onto LB agar plates. The plates were dried, inverted, and incubated overnight at 37°C. The number of viable bacteria was counted the next day.

For the growth curve analysis, once the bacterial culture reached an OD_600_ of approximately 0.4, 100 μL of culture was transferred into 20 mL of fresh LB medium. L-arabinose was added to a final concentration of 0.2%, and the culture was incubated in a constant temperature shaker at 37°C and 220 rpm. OD_600_ was measured at hourly intervals, and the data was used to generate a growth curve.

### Microscopy and image acquisition

Fluorescence image visualization of the Z ring used a mutant strain FtsZ-G55-mNeonGreen-Q56, which replaced genomic *ftsZ* with *ftsZ-mNeonGreen* under its normal genomic control (Moore et al., 2017). After the plasmid pBAD22a carrying the *icd* gene was transferred into the strain, the bacteria were cultured in LB medium with 100 μg/mL carbenicillin until the OD_600_ reached ~0.4, followed by the addition of 0.2% arabinose for induction at 37°C for 3 h. A droplet with 10 μL of bacterial cells was placed onto an agarose spacer, and the cells were visualized using a Leica DMI3000B fluorescence microscope. A common light source was used to observe bacterial morphology, while a mercury lamp was employed for fluorescence observation, with an excitation wavelength of 488 nm for mNeonGreen. The lengths of individual cells were measured, and the results are presented as mean ± SD.

DAPI staining of bacterial nucleoids was performed using a stock solution of 20 mg/mL and a working concentration of 5 μg/mL. At room temperature (25°C), 1 mL of the working solution was applied to the bacterial cells for 8 min under dark conditions. The cells were then washed four times with PBS buffer (137 mM NaCl, 2.7 mM KCl, 10 mM Na_2_HPO_4_, 1.8 mM KH_2_PO_4_, pH 6.5). The cells were visualized using a Leica DMI3000B fluorescence microscope with an excitation wavelength of 360 nm for DAPI.

### Gene expression and protein purification

The pET28a-*His*-*ftsZ* plasmid was transformed into *E. coli* BL21, plated on kanamycin-containing agar plates (50 μg/mL), and incubated overnight at 37°C in a constant temperature incubator. Monoclonal clones were selected and inoculated into 100 mL of TY medium (2.4% yeast extract, 1.2% tryptone, 0.94% wt/vol K_2_HPO_4_, and 0.02% wt/vol KH_2_PO_4_) under oxic conditions, and cultured for 24 h at 37°C with shaking at 220 rpm. The cultures were centrifuged at 3000 g for 20 min at ambient temperature, with the supernatant discarded. The precipitate was resuspended in the buffer containing 50 mM Tris, 300 mM NaCl at pH 7.5, with 1% PMSF, 1% lysozyme, and 1% Triton X-100 added, and incubated on ice for 30 min. The cell disruption was performed at 1000 Bar until the lysate was clear and transparent. The lysate was centrifuged at 18,000 g for 20 min at 4°C, and the supernatants and pellets were collected and analyzed for protein production by SDS-PAGE.

The pME6032-*GST-icd* recombinant plasmid was transformed into PAO1 by electrotransformation, grown to an OD_600_ of approximately 0.6. Gene expression was induced with 0.8 mM IPTG at 16°C and 220 rpm for 24 h. Following cell lysis, the supernatant was analyzed by Western blot to examine protein production.

### Bacterial two-hybrid screen

Bacterial two hybrid screening has been modified based on previous reports (Karimova et al., 1998). The plasmids pUT18-*icd* and pKNT25-*ftsZ* were simultaneously electrotransformed into *E. coli* BTH101. The bacteria were cultured on solid medium containing 40 μg/mL X-gal (5-bromo-4-chloro-3-indolyl-*β*-D-galactoside) as a chromogenic substrate and 50 μg/mL kanamycin and 100 μg/mL carbenicillin as antibiotics. IPTG (isopropyl-β-D-thiogalactoside) was typically added to the medium at a concentration of 0.5 mM to induce the expression of the full hybrid protein and the β-galactosidase reporter gene. To exclude false-positive clones, putative positive clones were selected and reseeded on LB agar plates containing 50 μg/mL kanamycin, 100 μg/mL carbenicillin, 0.5 mM IPTG, and X-gal, with true positive clones expected to yield blue colonies on these plates once again.

Bacterial cells with β-galactosidase activity to be detected were added with 0.5 mM IPTG and appropriate antibiotics in 3–5 mL LB and stirred 6 h at 220 rpm at 37°C. 50 μL bacterial suspension was added to 370 μL of Z buffer (100 mM Na_2_HPO_4_, 10 mM NaH_2_PO_4_, 10 mM KCl, 1 mM MgSO_4_, 5.4 μL/mL β-galactosidase, pH 7.0), followed by the addition of 20 μL chloroform and 10 μL of 0.1% SDS solution. After incubation on ice for 30 min, 4 mg/mL ONPG (o-Nitrophenyl β-D-galactopyranoside) was added, and the mixture was well mixed before being placed at 30°C for the reaction. After 30 min, a yellow color was observed. The reaction was terminated by adding 250 μL of 1 M Na_2_CO_3_ solution, and the time of color change was accurately recorded. Absorbance at 420 nm and 550 nm was measured. Miller Units were calculated using the formula: Miller Units = 1,000 x (OD_420_–1.75 x OD_550_)/t(min) x V(mL) x OD_600._

### *In vitro* pull-down analysis

After lysing the *P. aeruginosa* PAO1 strain overexpressing *GST-icd* or the *E. coli* BL21 strain overexpression *His-ftsZ*, the samples were centrifuged at 18000 g for 20 min, and the supernatants were collected separately. After mixing the supernatants containing 1 mL of GST-Icd and 50 μL of His-FtsZ, the mixture was added to the cleaned 50 μL GST resin and incubated for 2 h then washed 5 times with PBS buffer. The mixture of GST protein and His-FtsZ protein, as well as a supernatant containing only GST-Icd or His-FtsZ were used as controls. After the protein bound to the resin, the resin was centrifuged at 3000 g for 2 min, and the supernatant was discarded. The samples were washed three times with 1 mL PBS buffer, and the final centrifugation step ensured maximum removal of the supernatant.

The samples were checked by western blotting. Proteins first were separated using SDS-PAGE gel electrophoresis and transferred from the gel to polyvinylidene fluoride (PVDF) membranes at 220 mA for 90 min and then blocked with 5% skim milk in TBST for 1 h at room temperature on a shaker. The membranes were incubated with primary antibodies overnight at 4°C, washed three times with TBST for 10 min each, and then incubated with secondary antibodies, an HRP-conjugated Goat Anti-Rabbit, for 1 h at room temperature. The membranes were washed three times with TBST for 10 min. The results of Western Blots were detected using the ECL Plus kit (Sangon Biotech). Luminol and hydrogen peroxide in the kit were mixed at a ratio of 1:1 and applied to the PVDF membrane. The membrane was then placed in the dark chamber of the chemiluminescent imaging system and exposed for 10 s to obtain the images. The ECL developing solution reacted with the HRP - labeled antibody to produce a fluorescent signal with a maximum emission wavelength of 425 nm. The primary antibodies used were Anti-GST tag and Anti-His tag antibodies, while the secondary antibody was HRP-conjugated Goat Anti-Rabbit. All antibodies were sourced from Shanghai Sangon Inc.

## Result

### Expression of *icd* inhibits *E. coli* division

**Figure 1:**
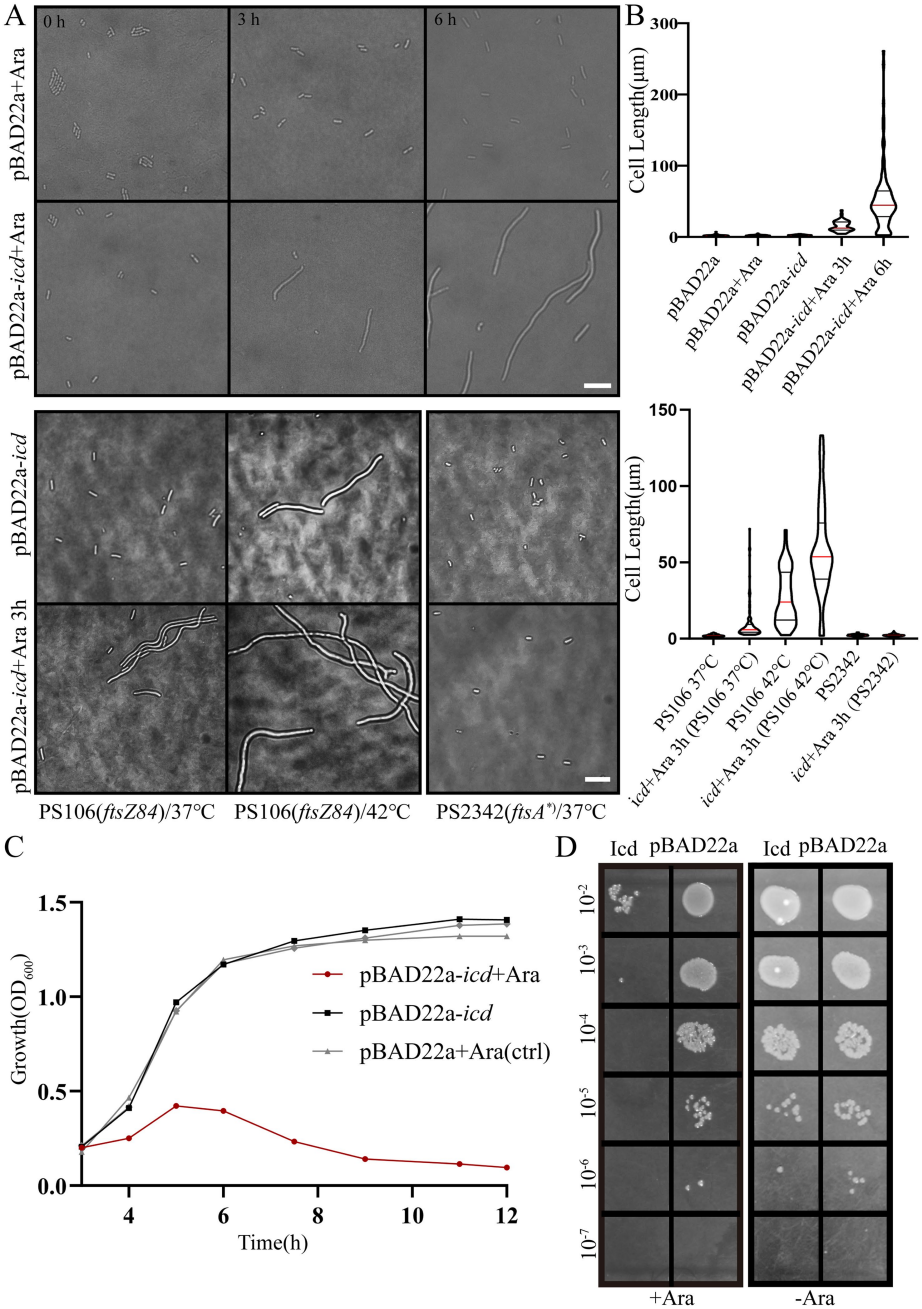
Induction of Icd interferes with *E. coli* cell growth and division. **(A)** Microscope images of *E. coli* DH5α*, E. coli* PS106 *(ftsZ84)* and PS2342 (ftsA*) after overexpression of *icd* for 3 and 6 h. Scale bar: 10 μm. **(B)** The violin plot shows the bacterial length after adding 0.2% L-arabinose 3 and 6 h, with the red line indicating the median and the black line representing the quartiles. **(C)** The growth curve of *E. coli* shows that overexpression of *icd* inhibits bacterial growth. **(D)** Serial spot dilutions assay shows that the number of viable bacteria decreased significantly after 6 h of overexpression of *icd*.

Overexpression of *icd* has a serious impact on the growth of *E. coli*. When we introduced the pET plasmid fused with the *icd* fragment into *E. coli* strain BL21 or C41, the bacteria had difficulty forming clones and growing in liquid culture, possibly due to leakage production of the protein. After the pET28a-*GST-icd* plasmid was transformed into *E. coli* strain BL21 or C41, it took almost 48 h to form visible colonies on the plate, furthermore, when the bacteria in a single colony were transferred to liquid LB medium, the bacteria hardly grew. To study the effect of Icd on *E. coli* division, we selected the pBAD22a plasmid with stricter arabinose-inducible expression control, constructed the recombinant plasmid pBAD22a-*icd*, and transformed it into *E. coli* DH5α. Overexpression of *icd* induced by 0.2% L-arabinose resulted in severe filamentation formation of *E. coli*. The average length of bacteria was 15 ± 1.1 μm after 3 h of overexpression and 53 ± 4.9 μm after 6 h of overexpression (Figures 1A,B).

To further investigate this, we examined the sensitivity of two mutant strains, PS106 *(ftsZ84)* and PS2342 (ftsA*), to Icd. PS106 *(ftsZ84)*, a *ftsZG105S* mutant, is a temperature-sensitive mutation that causes *E. coli* cells to divide severely defectively and grow into filaments at the restrictive temperature (42°C), whereas they grow normally at 37°C. This mutation FtsZG105S protein is impaired in GTP binding and GTPase, as well as in its polymerization (RayChaudhuri and Park, 1992; RayChaudhuri and Park, 1994). Another *E. coli* mutant, PS2342 (ftsA*), is a mutant of *ftsA*. Compared to wild-type FtsA, the FtsA* (*ftsAR286W*, a *zipA*-bypass *ftsA* mutant allele) interacts more strongly with FtsZ than FtsA, accelerating the turnover of FtsA* in the ring (Geissler et al., 2007).

As shown in Figures 1A,B, Icd still inhibited cell division in PS106 *(ftsZ84)*, whereas PS2342 (FtsA*) was resistant to Icd. PS106 grew normally at 37°C with an average length of 2.04 ± 0.04 μm. After 3 h of *icd* gene expression, the average length of PS106 increased to 10.1 ± 1.24 μm. At 42°C, PS106 had already exhibited division defects (27.4 ± 1.7 μm), and *icd* expression exacerbated these defects, resulting in an average length of 59.2 ± 3.4 μm. Surprisingly, the expression of *icd* did not affect the growth of strain PS2342 (ftsA*). It is possible that FtsA* enhanced the polymerization of FtsZ, thereby reducing the inhibitory effect of Icd.

Growth curve analysis showed that *E. coli* overexpressing *icd* reached its maximum OD_600_ value after 5 h and was followed by a decrease in OD_600_ value, signaling possible bacterial death or degradation (Figure 1C). Viable counts indicated that Icd caused severe damage to *E. coli*, resulting in a 1,000-fold reduction in viable cell numbers (Figure 1D). The inhibition of bacterial growth by Icd protein was species-specific. When the pME6032-*icd* recombinant plasmid was introduced into *P. aeruginosa* and the *icd* was induced for expression, it had no significant effect on growth (Supplementary Figure S1).

### Icd affects the localization of *E. coli* FtsZ

Due to the overexpression of *icd* leading to bacterial elongation, which may indicate inhibition of bacterial division, we then investigated the effect of *icd* expression on the formation of Z-ring, the bacterial divisome. Here, we used the *E. coli* mutant strain, FtsZ-G55-mNeonGreen-Q56 strain (Moore et al., 2017), in which the fluorescent protein mNeonGreen was inserted between positions 55 and 56 of FtsZ to replace the original FtsZ, to observe the Z-ring localization.

**Figure 2:**
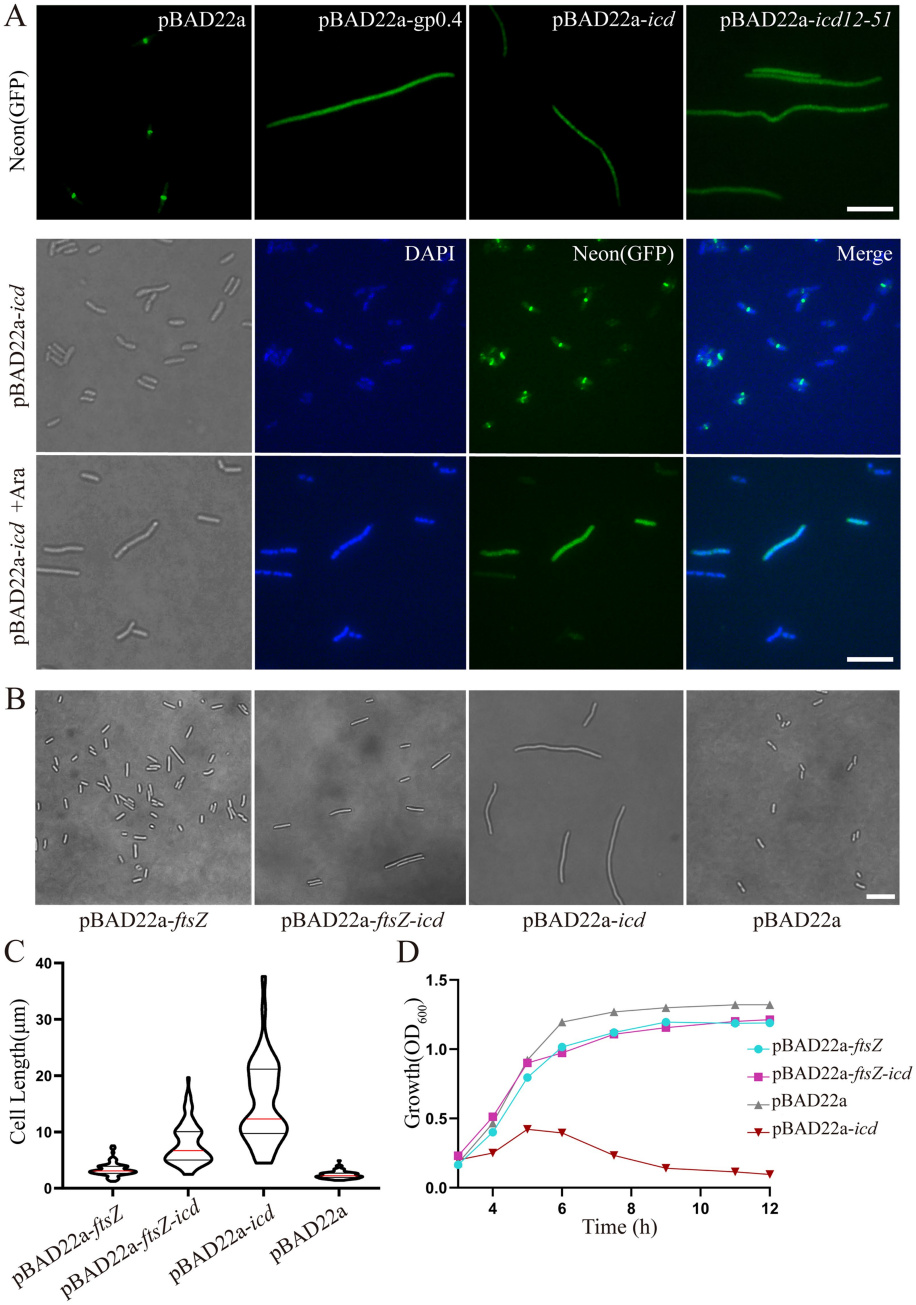
**(A)** Fluorescence microscopy images showed that *icd* overexpression blocked Z-ring formation. The *E. coli* mutant strain, FtsZ-G55-mNeonGreen-Q56 strain, was used to observe the Z-ring localization. When *icd* was overexpressed, FtsZ-mNeonGreen diffused in the cell, which is similar to the overexpression of FtsZ inhibitory protein GP0.4. Scale bar: 10 μm. DAPI staining was used to visualize the bacterial nucleoid. Under the influence of Icd, it was observed that bacterial division was inhibited, but the nucleolus structure remained intact and unaffected. **(B)** Comparison of microscopic images of *E. coli* DH5α overexpressing *ftsZ*, *icd* or co-overexpressing of *icd* and *ftsZ* for 3 h. Scale bar: 10 μm. **(C)** The violin plots of the bacterial length, where the red line indicates the median and the black line represents the quartile. **(D)** The growth curve results further show that the simultaneous overproduction of FtsZ reduced the inhibitory effect of Icd on bacteria.

FtsZ-mNeonGreen was always located in the middle of the cell, indicating that the Z-ring was assembled. After the pBAD22a-*icd* plasmid was transferred, the cell appeared filamentous and FtsZ-mNeonGreen diffused throughout the cell, denoting that Z-ring was not assembled correctly. It has been reported that protein Gp0.4 directly inhibited FtsZ assembly in *E. coli* (Kiro et al., 2013), therefore, we used *E. coli* with a plasmid overexpressing *gp0.4* as a control. We could observe the same phenomenon with overexpression of *gp0.4* or *icd* (Figure 2A). We preliminarily speculate that Icd may directly or indirectly act on FtsZ or Z-ring binding proteins in *E. coli*.

To determine whether Icd affects bacterial DNA replication, DAPI staining was used to visualize the bacterial DNA. The results showed that although cell division was inhibited, DNA replication remained intact and unaffected (Figure 2A).

We also constructed the recombinant plasmid pBAD22a-*icd*-*mCherry* and introduced it into *E. coli* FtsZ-G55-mNeonGreen-Q56. After induction with a low concentration of arabinose (0.02%) for 0.5 h, both Icd-mCherry and FtsZ-mNeonGreen were unable to be located correctly and instead dispersed throughout the entire bacteria (data not shown).

Overexpression of *ftsZ* simultaneously could reduce the inhibitory effect of Icd (Figures 2B–D). After 3 h of simultaneous overexpression of *icd* and *ftsZ* using plasmid pBAD-*ftsZ-icd* and 0.2% arabinose, the length of the bacteria became longer, but was significantly shorter than that of the strain overexpressing only *icd* using plasmid pBAD22a-*icd* (Figure 2B). The average cell length was 7.8 ± 0.39 μm, which was shorter than the 15 ± 1.1 μm of a strain overexpressing *icd* alone (Figure 2C). The results of the *E. coli* growth curve further confirmed that simultaneous overexpression of *ftsZ* significantly weakened the inhibitory effect of *icd* (Figure 2D). When *icd* was overexpressed, the OD_600_ value slowly increased in the first 5 h and then began to decrease. When *ftsZ* and *icd* were overexpressed simultaneously, the OD_600_ value continued to increase and reached a maximum value at around 8 h.

### Bacterial two-hybrid and pull-down experiments identified the interaction between Icd and FtsZ

The bacterial two-hybrid system is an effective method for studying the protein–protein interactions *in vivo* (Karimova et al., 1998). We recombinantly fused Icd to the T18 fragments of the adenylate cyclase, FtsZ to the T25 fragments, and transformed them into *E. coli* BTH101. We used pKNT25 and pUT18C empty plasmids as negative controls and pKNT25-*zip* and pUT18C-*zip* plasmids as positive controls. Thus, the Zip proteins could polymerize to form a dimer, resulting in *β*-galactosidase activity in the bacteria. Additionally, co-transformations of pKNT25-*ftsZ* with the pUT18C empty plasmid and pUT18C-*icd* with the pKNT25 empty plasmid into *E. coli* BTH101 served as two other negative controls (data not shown).

**Figure 3:**
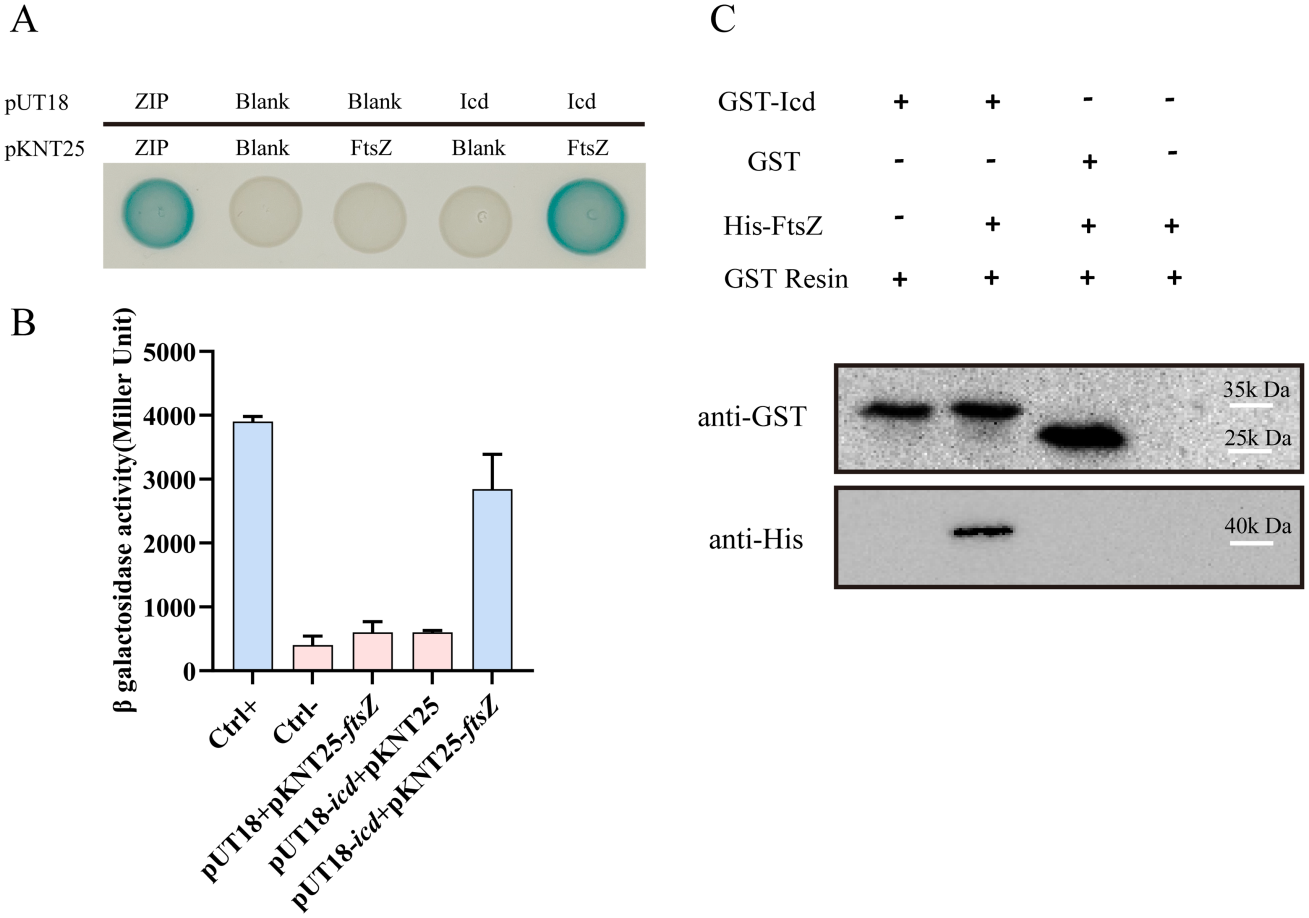
The interaction between FtsZ and Icd was confirmed by bacterial two-hybrid and pull-down assay. **(A)** Bacterial two-hybrid assay results showed the FtsZ-Icd interaction. When pKNT25-*ftsZ* and pUT18C-*icd* plasmids were transferred into the *E. coli* BTH101 strain, bacterial colonies appeared blue. All three negative controls (Ctrl-) were white spots. **(B)** The bar graph shows the statistical plot of *β*-galactosidase activity, which clearly shows the interaction between FtsZ and Icd. **(C)** Pull-down assay further validated the interaction between FtsZ and Icd. A solution containing GST-Icd was mixed with a His-FtsZ sample, separated with GST resin, and shown as an immunoblot against GST and His-tag in the cell extract. GST-Icd alone and a mixture of GST tag and FtsZ were used as controls. The results showed GST-Icd could interact with the His-FtsZ protein.

After the recombinant plasmid was transformed into *E. coli* BTH101 and cultured on solid medium containing IPTG and X-gal (5-bromo-4-chloro-3-indolyl-β-D-thiogalactopyranoside) for 26 h, most of the bacteria cells containing pKNT25-*ftsZ* and pUT18C-*icd* turned blue, while the negative control group remained white (Supplementary Figure S2). Figure 3A shows a single clone randomly selected from the initial culture medium, in which the cells containing pKNT25-*ftsZ* and pUT18C-*icd* and the positive control containing pKNT25-*zip* and pUT18C-*zip* were blue, while the negative control strain was white. The activity of β-galactosidase was measured by UV–VIS spectrophotometer to quantify the results of the bacterial two-hybrid measurement, further confirming that Icd and FtsZ interacted in the cells (Figure 3B).

A pull-down assay was also conducted to confirm the interaction between Icd and FtsZ. Since Icd inhibited *E. coli* growth, the production level of Icd protein expressed in *E. coli* was low, therefore, we used *P. aeruginosa* PAO1 and the recombinant plasmid pME6032-*GST-icd* to overexpress Icd protein. The cell lysates containing Icd protein were purified and enriched by GST resin, and mixed with His-FtsZ sample. We used a mixture of GST protein and His-FtsZ as a control. After elution and immunoblotting with anti-GST and anti-His antibodies, it was found that GST-Icd could pull down His-FtsZ, while the GST tag alone could not (Figure 3C). This suggested that Icd interacts directly with FtsZ.

### The antibacterial core region of Icd was around 12–51

**Figure 4:**
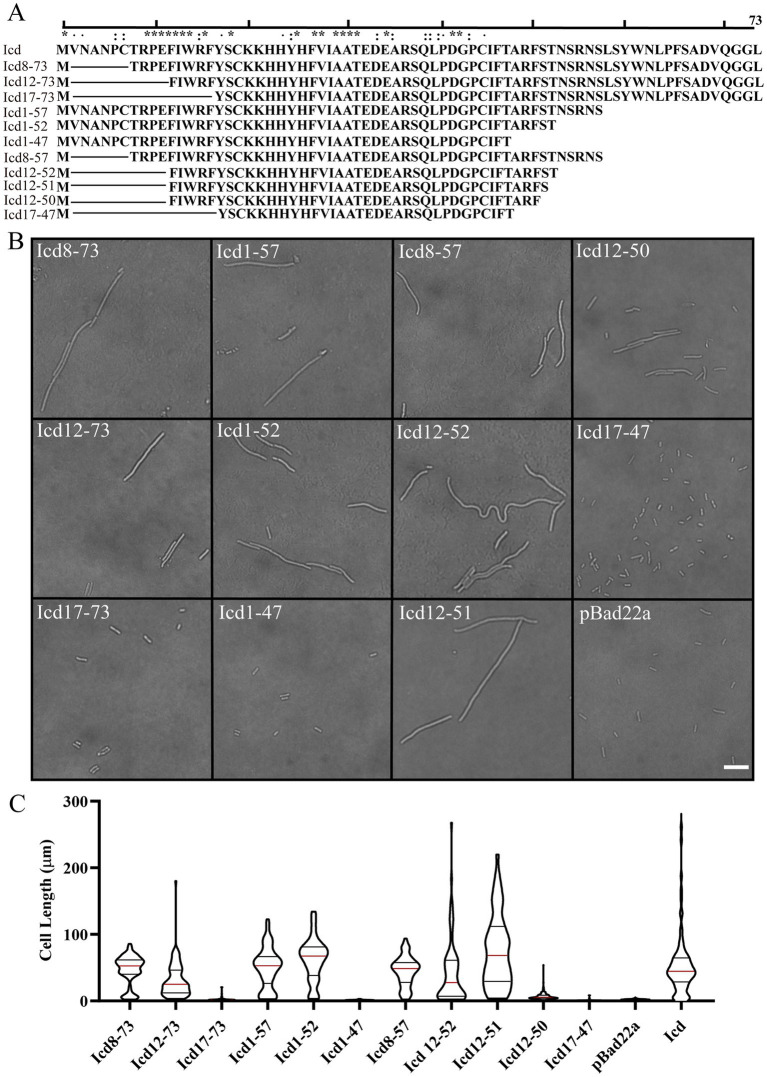
Effects of overexpression of *icd* mutant genes of different lengths on bacterial cell length. **(A)** The list of constructed Icd mutant proteins of different lengths. **(B)** Microscopic image of the effect of overexpression of *icd* mutant genes of different lengths on bacterial growth. The scale bar is 10 μm. **(C)** The violin plots summarize the statistical values of bacterial lengths after overexpression of *icd* of different lengths, with the red line indicating the median and the black lines indicating the quartiles.

Amino acid sequence alignment revealed that Icd proteins exist not only in the P1 phage, but also in some other Enterobacteriaceae phages, although there are some sequence differences (Supplementary Figure S3). Based on the alignment, we constructed recombinant plasmids containing various lengths of *icd* gene deletions and determined the antibacterial core sequence region of Icd by observing bacterial growth. Icd sequences of different lengths are listed in Figure 4A. Icd sequences of different lengths were inserted into the pBAD22a plasmid and transformed into *E. coli* DH5α.

Firstly, we observed the effect of mutated Icd on bacterial length under a microscope (Figure 4B). We found that when the N-terminal truncation of Icd proteins exceeds 11 amino acids or the C-terminal exceeds 24 amino acids, the overexpressed *icd* mutant gene loses its antibacterial effect. Among the Icd mutants we constructed, Icd12-51 appeared to be the shortest protein with the same ability to inhibit bacterial division as full-length Icd. After overexpressing *icd12-51* for 6 h, the average length of *E. coli* reached 48 ± 5.5 μm, and the longest bacteria we observed reached 220 μm. However, the average length of bacteria overexpressing *icd12-50* decreased to 7 ± 0.34 μm, indicating a significant reduction in inhibitory ability (Figures 4B,C).

**Figure 5:**
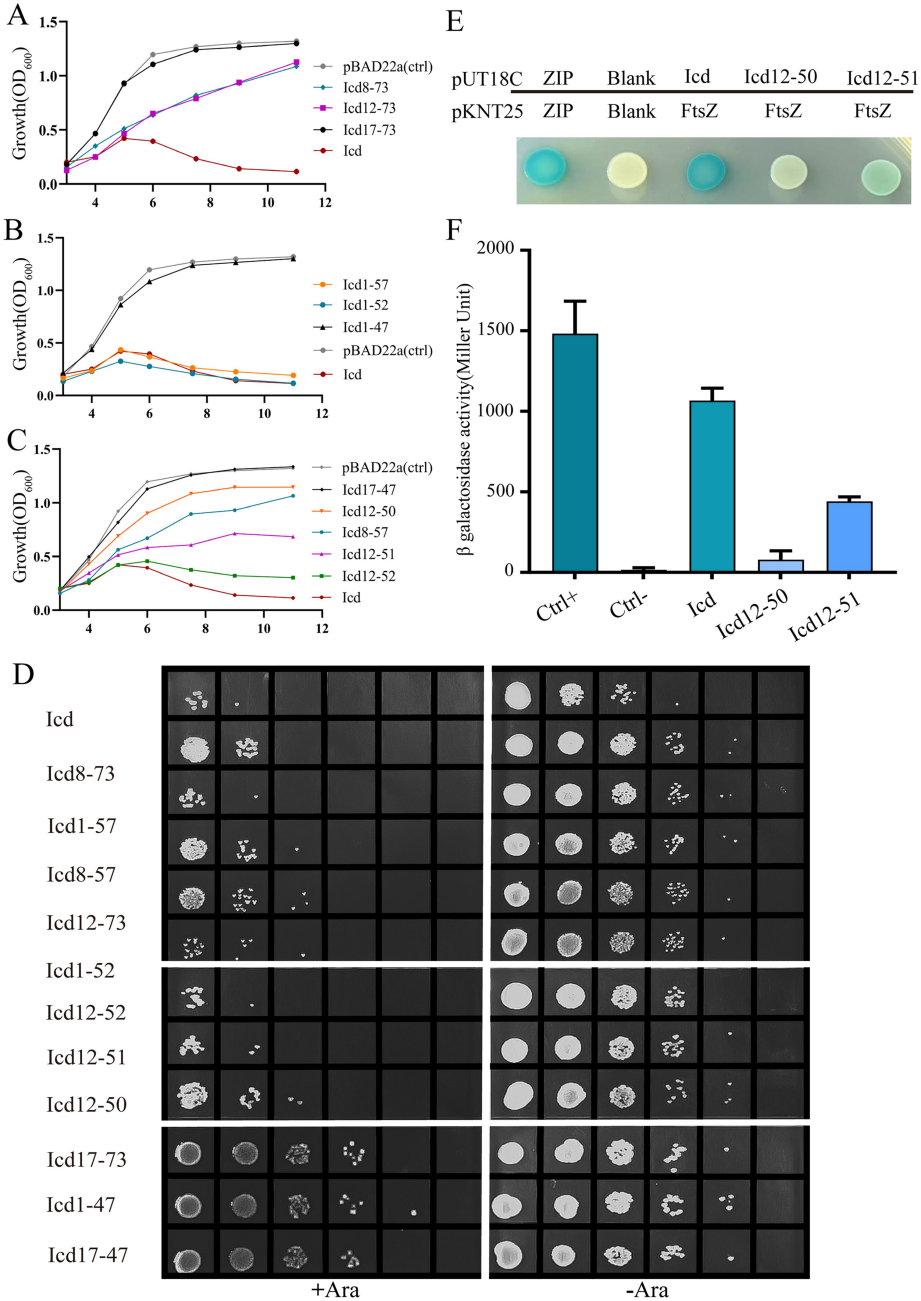
The bacterial growth curve further confirmed that the antibacterial core region of the Icd protein was 12–51. **(A–C)** Bacterial growth curve illustrating the effects of Icd of different lengths on bacterial growth. **(D)** The number of viable cells was examined by serial dilution method after overexpression of *icd* mutant genes of different lengths for 6 h. **(E,F)** Bacterial two-hybrid assay results showed the FtsZ-Icd, FtsZ-Icd12-51 and FtsZ-Icd12-50 interaction. The statistical plot of β-galactosidase activity shows and Icd12-50, and the interaction of FtsZ-Icd12-51 is weaker than that of FtsZ-Icd, while the interaction between icd12-50 and FtsZ is even weaker.

Next, we measured the bacterial growth curves to test their antibacterial activity. Overexpression of *icd* genes has a strong inhibitory effect on bacterial growth, which we used as a positive control, and the growth of bacteria containing an empty pBad22 plasmids as a negative control. The OD_600_ values of *E. coli* overexpressing *icd* genes increased slowly in the first 5 h, which may be due to an increase in bacterial numbers or the formation of filamentous bacteria. Afterwards, the OD_600_ value began to decrease and dropped to baseline after 9 h, indicating bacterial death (Figures 5A–C). Figure 5A examined the effect of overexpression of N-terminally truncated Icd mutant proteins on *E. coli* growth. The results showed that removing 7 or 11 amino acids from the N-terminus of the Icd mutant proteins still had partial inhibitory effects, while removing 16 amino acids from the N-terminus resulted in the disappearance of antibacterial activity. Figure 5B examines the effect of C-terminal truncation on the antibacterial activity of Icd proteins. Truncating the 21 amino acids at the C-terminus (Icd1-52) did not affect the antibacterial activity of the Icd protein. Figure 5C shows the antibacterial effect of Icd proteins with both N-terminal and C-terminal truncations to search for the shortest core region. The results showed that Icd12-52 had almost the same antibacterial activity as full-length Icd, with OD_600_ increasing slowly in the first 6 h then decreasing. Overexpression of *icd12-51* also had strong antibacterial activity, but no decrease in OD_600_ was observed, indicating that the antibacterial activity was partially weakened. At the same time, the antibacterial effect of overexpression of *icd12-50* was significantly weakened (Figure 5C). Interestingly, the antibacterial activity of overexpression of *icd12-52* or *icd12-51* was stronger than that of overexpression of *icd8-73*, *icd12-73* or *icd8-57*, suggesting that the extra amino acids may weaken the antibacterial activity.

Not only does an increase in the number of bacteria cause an increase in OD_600_ value, but a significant increase in bacterial length will also increase OD_600_ value. To obtain more accurate results, we used the serial spot dilution assay to measure the number of viable bacteria and assess the antibacterial activity of Icd mutant proteins. Here, we measured the viable counts after 6 h of overexpression of *icd* and its mutant genes (Figure 5D). In the absence of arabinose, the number of *E. coli* colonies was nearly identical, around 4 × 10^8^ CFU/mL. When *icd* was overexpressed, the number of *E. coli* colonies decreases to 2 × 10^5^ CFU/mL. Meanwhile, the number of colonies overexpressing *icd12-52* or *icd12-51* was 2 × 10^5^ CFU/mL and 4 × 10^5^ CFU/mL, respectively. On the other hand, the colony count of overexpressed *icd12-50* was only 4 × 10^6^ CFU/mL, which was significantly weakened. In addition, the measurable viable counts after overexpression of *icd8-73, icd8-57 or icd12-73* were 2 × 10^6^, 2 × 10^6^ and 6 × 10^6^ CFU/mL, respectively, further demonstrating that their antibacterial activity was lower than that of *icd12-51*. The colony counts after overexpression of *icd12-52* and *icd12-51* were in the same order of magnitude, which concluded that Icd12-51 can be considered as the shortest core region with antibacterial effects similar to those of the full-length Icd.

Bacterial two-hybrid experiments confirmed the interaction between Icd12-51 and FtsZ (Figures 5E,F; Supplementary Figure S2). Most of the bacteria cells containing pKNT25-*ftsZ* and pUT18C-*icd12-51* turned blue, but the color was lighter than that of bacteria containing pKNT25-*ftsZ* and pUT18C-*icd*. Moreover, the *β*-galactosidase activity was only 40% of that of the bacterial cells containing pKNT25-ftsZ and pUT18C-icd (Figures 5E,F). It can be inferred that Icd12-51 forms an effective but slightly weaker interaction with FtsZ. Meanwhile, the cells containing pKNT25-*ftsZ* and pUT18C-*icd12-50* turned a lighter blue, and the β-galactosidase activity was approximately 12% of that of the cells containing pKNT25-*ftsZ* and pUT18C-*icd* (Figures 5E,F). This showed that Icd12-50 was still able to form a weaker interaction with FtsZ.

## Discussion

The co-evolution between phages and hosts has enabled phages to develop a variety of special ways to inhibit the growth of host bacteria, including numerous small proteins, which can help phages effectively utilize host cell resources. Bacterial division is one of the important processes of bacterial growth and an important target for inhibition. Inhibiting host division may prevent host daughter cells from escaping and maximize the utilization of host resources (De Smet et al., 2021); it may also delay bacterial lysis, allowing bacteria to grow larger and helping phages produce more offsprings (Dhanoa et al., 2022).

In this study, we determined that the small protein Icd of P1 phage can directly target the key bacterial division protein FtsZ, thereby inhibiting the growth and division of the host *E. coli*. We also concluded that Icd12-51 was the core region, and it has nearly the same antibacterial effect as the full length of Icd. This 40-aa small peptide strongly inhibits bacterial division, and its production can lead to severe bacterial filamentation.

**Figure 6:**
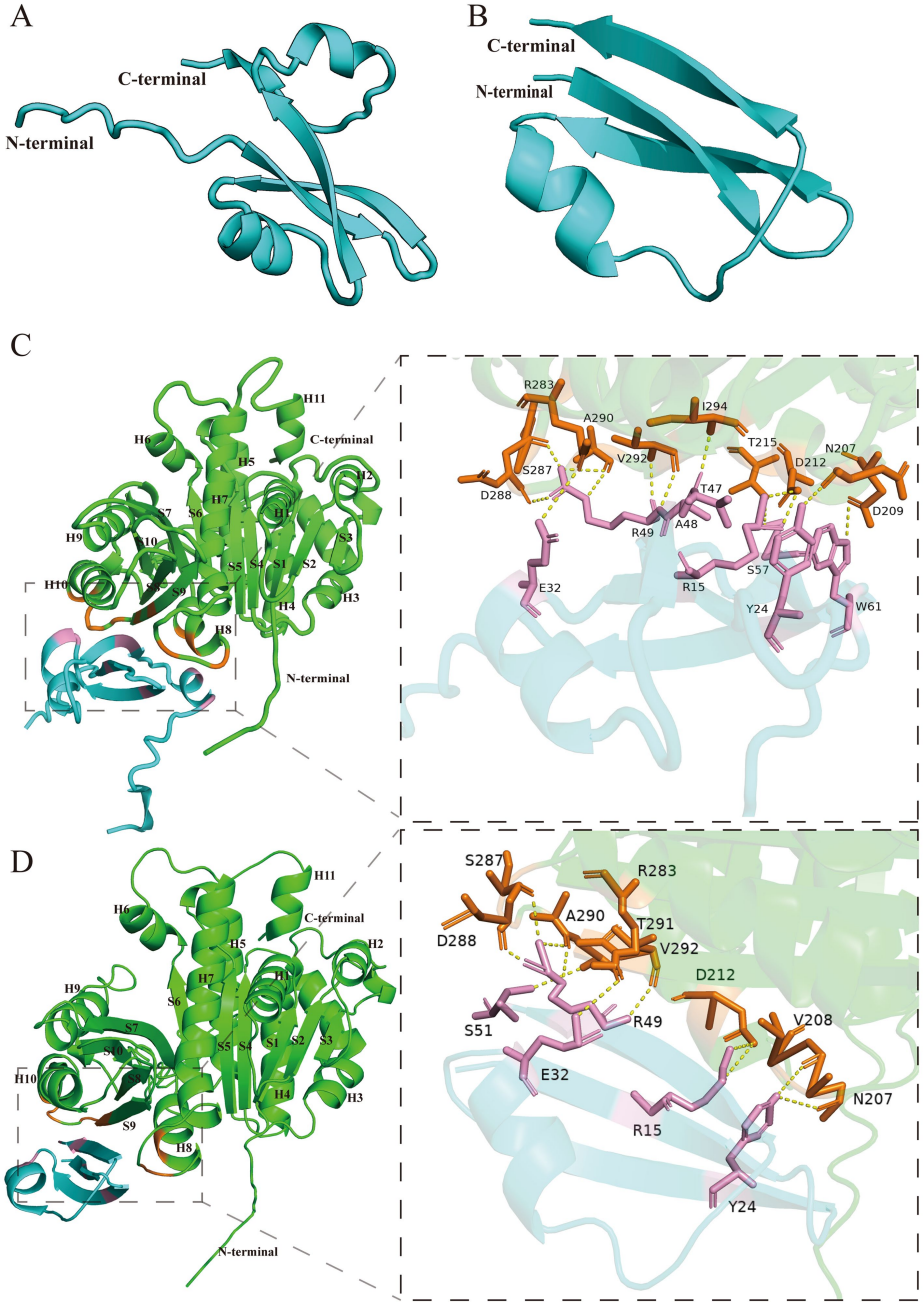
Structure prediction of Icd and the interactions between Icd and FtsZ. **(A)** Icd. **(B)** Icd12-52. **(C)** The interaction between Icd and FtsZ. The interaction interface is magnified inside the box. **(D)** The interaction between Icd12-51 and FtsZ. The interaction interface is magnified inside the box.

As we were unable to obtain high-quality protein for biochemical and structural studies, we used AlphaFold (Jumper et al., 2021) and AlphaFold3 (Abramson et al., 2024), which have advantages in the analysis of protein structure and protein–protein interaction, to predict the structure of Icd and its binding to FtsZ. The structure of Icd is bow-tie-shaped, including two small ɑ-helix and four *β*-sheets (Figure 6A). After deleting a small ɑ-helix and a β-sheet, its main structure Icd12-51 contains only a small ɑ-helix and three β-sheets (Figure 6B). FtsZ from *E. coli* is a globular protein consisting of 383 amino acids, with two subdomains, N terminus (amino acids 12–195) and C terminus (amino acids 196–316) connected by the H7 helix (Oliva et al., 2004; Chen and Erickson, 2011). To predict the binding sites of Icd and FtsZ, the amino acid sequences of FtsZ and Icd or Icd12-51 were input into AlphaFold 3 to analyze the possible binding sites. We found that different Icd sequence inputs resulted in slightly different binding modes. The results showed that Icd bound to the T7-H8 region and the H10-S9 region of FtsZ, and 13 hydrogen bonds could be formed between Icd and FtsZ (Figure 6C). At the interaction interface, residues N207, D209, D212 and T215 of FtsZ interact with multiple amino acids including W61, Y24, S57 and R15 of Icd, and residues R283, S287, D288, A290, V292, and I294 of FtsZ form 5 hydrogen bonds with E32, R49, A48 and T47 of Icd. In contrast to Icd, Icd12-51 truncated one ɑ-helix and one β-sheet, and three β-sheets and a small ɑ-helix were retained. The predicted binding sites of Icd12-51 to FtsZ are somewhat different from those of Icd-FtsZ, but are still located in these two regions of FtsZ (Figure 6D). The predicted binding sites of Icd12-51 to FtsZ indicated that N207, V208 and D212 of FtsZ could form hydrogen bonds with Y24, R15 of Icd and R283, S287, D288, A290, T291, V292 of FtsZ interacts with E32, R49 of S51 of Icd. These predictions are consistent with our bacterial two-hybrid results. Icd12-51 is sufficient to interact with FtsZ, but its interaction is slightly weaker than that of Icd with FtsZ. The N207, D209 and D212 of FtsZ are located on the T7 loop and H8 helix, the most important amino acid sites related to FtsZ polymerization and GTPase activity. Occupancy of these sites will strongly inhibit the polymerization ability of FtsZ, which can explain why Icd has a strong antibacterial effect.

Studies searching for antibacterial proteins in phages have also reported several different types of small proteins in different phages that can specifically target FtsZ and inhibit bacterial division, such as Kil in phage *λ* (Haeusser et al., 2014; Hernandez-Rocamora et al., 2015), GP0.4 in phage T7 (Kiro et al., 2013; Simpkin Adam and Rigden, 2016) and Hdi in phage T5 (Mahata et al., 2023). All of them would lead to abnormal FtsZ localization in cells, preventing the Z-ring formation and causing cell filamentation. However, their mechanism of inhibiting FtsZ differed from that of Icd proteins.

Even though these proteins have little sequence similarity, the three small proteins Kil, GP0.4 and Hdi appear to have similar structures (Mahata et al., 2023). They all contain mainly two ɑ-helices, forming a U-shaped or V-shaped helix-turn-helix structure. Previous studies suggested that they may have similar FtsZ binding sites and inhibitory regions. This helix-turn-helix structure was different from the bow-tie-like structure of Icd. Simpkin Adam and Rigden (2016) analyzed the interaction between GP0.4 and FtsZ and predicted that the GP0.4 protein inserts into the cleft between helices 1, 5 and 7 of FtsZ. Among them, the conserved FtsZ residues G21, N24, G47, and G107 interact with GP0.4 through hydrogen bonds, thereby inhibiting the polymerization of FtsZ. Although these proteins and Icd share the function of inhibiting FtsZ activity, it was evident that Icd has different structures and binding sites with FtsZ.

Kiro et al. (2013), in their study of GP0.4 protein, found a random mutant FtsZ, a glycine-valine repeat sequence inserted at position 18 of FtsZ, resistant to the inhibition of GP0.4. This result supports this model. However, Haeusser et al. (2014) isolated the mutants FtsZ_V208A_ and FtsZ_L169R_, which could resist the inhibition of bacterial division by Mahata et al. (2023) found that both FtsZ_F268V_ and FtsZ_G191S_ were resistant to the inhibition of Hdi and GP0.4 in *E. coli*. These mutation sites are far from the binding site and do not seem to support this model. It is possible that these mutated FtsZ proteins affect the polymerization or bundling of FtsZ or affect the interaction with other association proteins, thereby reducing the inhibitory effect of these phage peptides on bacterial growth. Therefore, further research is still needed.

Interestingly, our results show that overexpression of *icd* does not inhibit bacterial division of ftsA* mutants, a *zipA*-bypass *ftsA* mutant allele. Considering protein FtsA* can enhance the polymerization of FtsZ (Geissler et al., 2007), it could resist the inhibitory effect of Icd. Correspondingly, Haeusser et al. (2014) found that another phage protein Kil could not inhibit the ftsA* strain after the knockout of ZipA, although it still inhibited the ftsA* strain. They believed that the inhibitory effect of Kil on FtsZ required the presence of ZipA. This is different from our experimental results. Subsequent biochemical studies found that the inhibitory effect of Kil on the polymerization of FtsZ does not require ZipA (Hernandez-Rocamora et al., 2015). Further studies are still needed to clarify these mechanisms.

In addition, searching for more antimicrobial proteins in phages has potential value for understanding phage-host interactions and screening new antimicrobial proteins to address the problem of multiple antibiotic resistance in pathogens.

## Data Availability

The original contributions presented in the study are included in the article/Supplementary material, further inquiries can be directed to the corresponding authors.
