# Supplementary material for: Bacteriophage P1 protein Icd inhibits bacterial division by targeting FtsZ
Source: Front Microbiol. 2025 Feb 26;16:1533694. doi: 10.3389/fmicb.2025.1533694 (PMC11897509; doi:10.3389/fmicb.2025.1533694)
Supplement: Supplementary file 1 [file Data_Sheet_1.pdf]

pME6032-*icd* pME6032

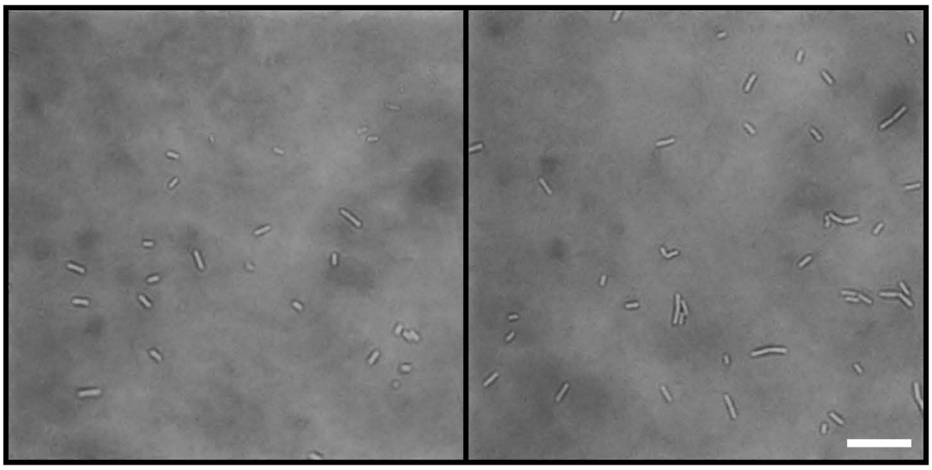

P. aeruginosa PAO1

Figure S1. Images of *icd* gene expression induced by transforming the PME6032-*icd* plasmid into the PAO1 strain.

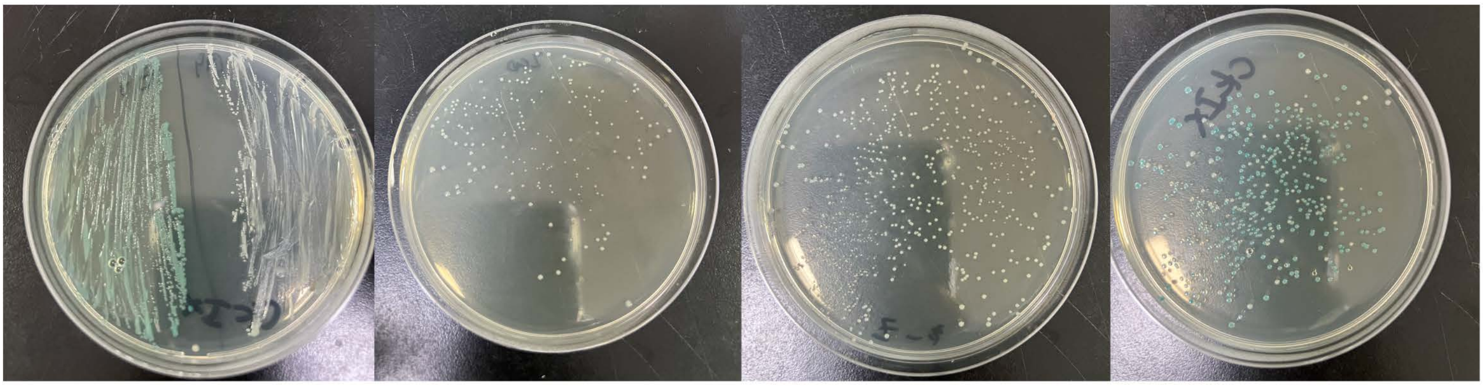

pUT18C-*zip* pKNT25-*zip* | pUT18C pKNT25 | pUT18C-*icd* pKNT25 | pUT18C pKNT25-*ftsZ* | pUT18C-*icd* pKNT25-*ftsZ*

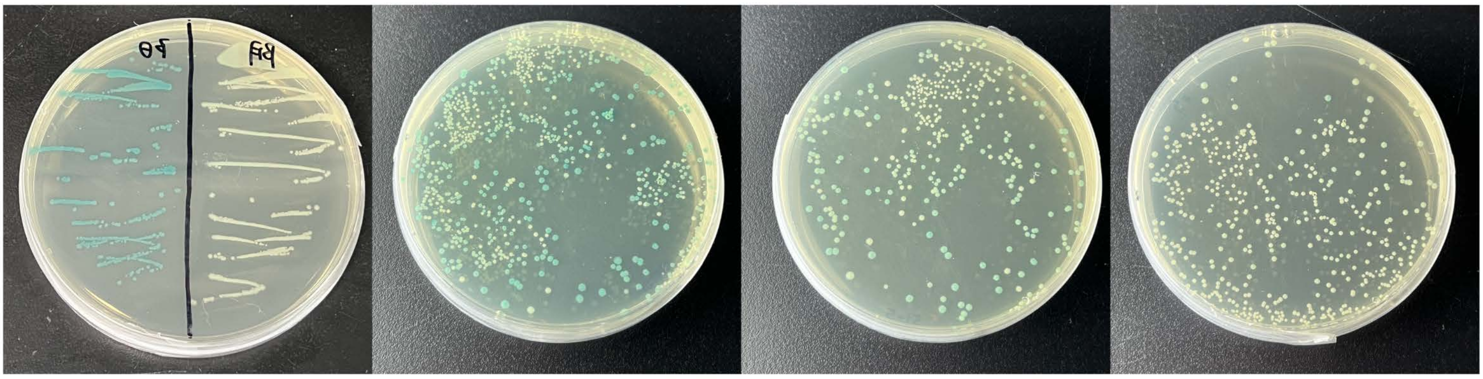

pUT18C-*zip* pKNT25-*zip* | pUT18C pKNT25 | pUT18C-*icd* pKNT25-*ftsZ* | pUT18C-*icd12-51* pKNT25-*ftsZ* | pUT18C-*icd12-50* pKNT25-*ftsZ*

Figure S2. Bacterial two-hybrid assays were used to verify the interaction between Icd and FtsZ. Bacteria were cultured on X-Gal, IPTG, and appropriate antibiotic plates. The positive control strain turned blue after 24 hours, while colonies in the Icd experimental group turned blue after 26 hours (approximately 80%). In the Icd12-51 group, around 50% of colonies turned blue, while only 10% of colonies in the Icd12-50 group showed blue color.

```
Escherichia_phage_D6-2                  MVNANPCSRPEFIWRFHSLQ-KHCDHFAIAATEKEARSLIPDQYGVLTGRFSTSPQPNTNPWYRNITNPGIAEAH   74
Escherichia_phage_D6                    MATIPTPTHSEFIWRFYSCQ-KHLYICVMAATESEARSYLPEEPCIFAARFTLDAMEILNYWNLPMNCVEVR---   71
Caudoviricetes_sp.-2                    MATTPTPTHSEFIWRFYSCQ-KHLYICVMAATESEARSYLPEEPCIFAARFTLDAMEILNYWNLPMNCVEVH---   71
Caudoviricetes_sp.-3                    MATIPTPTHSEFIWRFYSCQ-KHLYICVMAATEAEARSYLPEEPCIFAARFTLDAMEILNYWNLPMNCVEVH---   71
Escherichia_phage_P1                    MVNANPCTRPEFIWRFYSCK-KHHYHFVIAATEDEARSQLPDGPCIFTARFSTNSRNSLSYWNLPFSADVQGGL-   73
Bacteriophage_sp.                       MVNANPCARPEFIWRFYSCK-KHHYHFVIAATEDEARSQLPDGPCIFTARFSTDSRNSLSYWNLPFSADVQGGL-   73
Bacteriophage_sp.-4                     MVNANPCARQEFIWRFYSCSKKHHYHFVIAATEDEARSQLPDGPCIFTARFSTNSRNSLSYWSLPFSADVQGGL-   74
Bacteriophage_sp.-3                     MVNANPCARQEFIWRFYSCK-KHHYHFVIAATEDEARSQLPDGPCIFTARFSTNSRNSLSYWNLLFSADVQGDL-   73
Bacteriophage_sp.-2                     MVNANPCAHQEFIWRFYSCK-KHHYHFVIAATEDEARSQLPDGPCIFTARFSTNSRNSLSYWNLPFSADVQGDL-   73
Escherichia_phage_JL22                  MVNANPCARQEFIWRFYSCK-KHHYHFVIAATEDEARSQLPDGPCIFTARFSTNSRNSLSYWNLPFSADVQEVL-   73
Punavirus_P1                            MVNANPCARQEFIWRFYSCK-KHHYHFVIAATEDEARSQLPDGPCIFTARFSTNSRNPLSYWSLPFSADVQGGL-   73
Bacteriophage_sp.-1                     MVNANPCARQEFIWRFYSCK-KHHYHFVIAATEDEARSQLPDGPCIFTARFSTNSRNSLSYWNLPFSADVQGVL-   73
Enterobacteria_phage_P7                 MVNANPCARQEFIWRFYSCK-KHHYHFVIAATEDEARSQLPDGPCIFTARFSTNSRNSLSYWNLPFSADVQGGL-   73
Escherichia_phage_RCS47                 MVNANPCARQEFIWRFYSCK-KHHYHFVIAATEDEARSQLPDGPCIFTARFSTNSRNSLSYWSLPFSADVQGGL-   73
Escherichia_phage_vB_EcoM-Ro157c2YLVW   MVNANPCARPEFIWRFYSCQ-KGHYHFVIAPTEDEARSQLPDAPCIFSARFSTDLRNSLSYWCLPVNASAQEGL-   73
Escherichia_phage_vB_EcoM-673R5         MATIPTPTHPEFIWRFYSCQ-KRHYHFVIAPTEDEARSQLPDAPCIFSARFSTDSRNSLSYWCLPVNASAQEGL-   73
Bacteriophage_sp.-5                     MATIPTPAHPEFIWRFYSCQ-KRHYHFVIAPTEDEARSQLPDAPCIFSARFSTDSRN------------------   56
                                        *..    :: ******:* . *     .:* ** **** :*:   :::.**: .
```

Figure S3. NCBI sequence alignment results showed that Icd-like proteins are also found in other bacteriophages.

Table S1. List of strains and plasmids used in this study.

Strains and plasmids	Phenotype	Source or reference
Escherichia coli		
DH5α	*F φ80lacZ ΔM15 Δ(lacZYA-argF)U169 recA1 endA1 hsdR17(rk^{-}, mk)phoA supE44 thi-1 gyrA96 relA1 tonA^{+}*	Lab stock
BL21(DE3)	*F^{-} ompT gal dcm lon hsdSB*(rB- mB-) λ(DE3)	Lab stock
BTH101	*F0 , cya-99, araD139, galE15, galK16, rpsL1 (StrR), hsdR2, mcrA1, mcrB1, relA1 (the relA1 mutations is not indicated in the Euromedex manual.*	Lab stock
FtsZ-G55-mNeonGreen-Q56	BW27783-FtsZ-G55-mNeonGreen-Q56	Lab stock
PS106	W3110,Leu::Tn10 *ftsZ84*	Wuhan University
PS2342	W3110, Leu::Tn10 *ftsA**	Wuhan University
**Plasmid**		
pET28a	His6-tag expression vector, Amp	Lab stock
pET28a-*ftsz*	*ftsZ* was cloned into pET28a between the NcoI and HindIII sites.	This study
pBAD22a	Expression vector, Amp	Lab stock
pBAD22a-*icd*	*icd* was cloned into pBAD22a between the EcoRI and HindIII sites.	This study
pBAD22a-*icd8-73*	*icd8-73* was cloned into pBAD22a between the EcoRI and HindIII sites.	This study
pBAD22a-*icd12-73*	*icd12-73* was cloned into pBAD22a between the EcoRI and HindIII sites.	This study
pBAD22a-*icd17-73*	*icd17-73* was cloned into pBAD22a between the EcoRI and HindIII sites.	This study
pBAD22a-*icd1-57*	*icd1-57* was cloned into pBAD22a between the EcoRI and HindIII sites.	This study

pBAD22a-*icd1-52*	*icd1-52* was cloned into pBAD22a between the EcoRI and HindIII sites.	This study
pBAD22a-*icd1-47*	*icd1-47* was cloned into pBAD22a between the EcoRI and HindIII sites.	This study
pBAD22a-*icd8-57*	*icd8-57* was cloned into pBAD22a between the EcoRI and HindIII sites.	This study
pBAD22a-*icd12-52*	*icd12-52* was cloned into pBAD22a between the EcoRI and HindIII sites.	This study
pBAD22a-*icd12-51*	*icd12-51* was cloned into pBAD22a between the EcoRI and HindIII sites.	This study
pBAD22a-*icd12-50*	*icd12-50* was cloned into pBAD22a between the EcoRI and HindIII sites.	This study
pBAD22a-*icd17-47*	*icd17-47* was cloned into pBAD22a between the EcoRI and HindIII sites.	This study
pBAD22a- *ftsz*	*ftsZ* was cloned into pBAD22a between the NcoI and HindIII sites.	This study
pBAD22a- *ftsz-icd*	pBAD22a containing *ftsz* and *icd* gene connected by Rbs	This study
pKNT25	Bacterial two-hybrid plasmid	Lab stock
pKNT25-*ftsz*	FtsZ was cloned into pKNT25 between the HindIII and XbaI sites.	This study
pUT18C	Bacterial two-hybrid plasmid	Lab stock
pUT18C-*icd*	*icd* was cloned into pUT18C between the XbaI and KpnI sites.	This study
Pseudomonas aeruginosa		
PAO1	Wild type	Lab stock
**Plasmid**		
pME6032	Shuttle plasmid, IPTG induction, tetracycline	Lab stock

Table S2. Primers used in this study.

Primer	Sequence (5' → 3'')	Restriction site
pBAD22a-*icd*-FW	AAAgaattcatgGTTAACGCTAACCCG	EcoRI
pBAD22a-*icd*-RV	AAAaagcttTTACAGACCACCCTGCAC	HindIII
pBAD22a-*icd8-73*-FW	AAAgaattcatgACTCGTCCGGAATTTATC	EcoRI
pBAD22a-*icd12-73*-FW	AAAgaattcatgTTTATCTGGCGTTTCTATTCT	EcoRI
pBAD22a-*icd17-73*-FW	AAAgaattcatgTATTCTTGCAAAAAACACCA	EcoRI
pBAD22a-*icd1-57*-RV	AAAgaattcTTAGTTACGGCTGTTGGTG	HindIII
pBAD22a-*icd1-52*-RV	AAAaagcttTTAGAAGCGCGCGGTGAA	HindIII
pBAD22a-*icd1-47*-RV	AAAaagcttTTAGGTGAAGATGCACGGG	HindIII
pBAD22a-*icd12-51*-RV	AAAaagcttTTAGCTGAAGCGCGCGGT	HindIII
pBAD22a-*icd12-50*-RV	AAAaagcttTTAGAAGCGCGCGGTGAA	HindIII
pBAD22a- *ftsz*-FW	AAAccatggTTGAACCAATGGAACTTACC	NcoI
pBAD22a- *ftsz* -RV	AAAaagcttTTAATCAGCTTGCTTACGC	HindIII
pKNT25-*ftsz*-FW	AAAaagcttgATGTTTGAACCAATGGAACT	HindIII
pKNT25-*ftsz* -RV	AAAtctagaATCAGCTTGCTTACGCAG	XbaI
pUT18C-*icd*-FW	AAAtctagagATGGTTAACGCTAACCCG	XbaI
pUT18C-*icd* -RV	AAAggtaccTTACAGACCACCCTGCAC	KpnI
pME6032-*GST*-*icd*-FW	AAAgaattcATGTCCCCTATACTAGGTTATTGG	EcoRI
